# The Role of Gut-Derived, Protein-Bound Uremic Toxins in the Cardiovascular Complications of Acute Kidney Injury

**DOI:** 10.3390/toxins14050336

**Published:** 2022-05-11

**Authors:** Pauline Caillard, Youssef Bennis, Isabelle Six, Sandra Bodeau, Saïd Kamel, Gabriel Choukroun, Julien Maizel, Dimitri Titeca-Beauport

**Affiliations:** 1 Department of Nephrology, Dialysis and Transplantation, Amiens Medical Center, Amiens 80054, France; caillard.pauline@chu-amiens.fr (P.C.); choukroun.gabriel@chu-amiens.fr (G.C.); 2 MP3CV Laboratory, UR UPJV 7517, University of Picardy Jules Verne, Amiens 80054, France; bennis.youssef@chu-amiens.fr (Y.B.); isabelle.six@u-picardie.fr (I.S.); bodeau.sandra@chu-amiens.fr (S.B.); kamel.said@chu-amiens.fr (S.K.); maizel.julien@chu-amiens.fr (J.M.); 3 Department of Clinical Pharmacology, Amiens Medical Center, Amiens 80054, France; 4 Department of Clinical Biochemistry, Amiens Medical Center, Amiens 80054, France; 5 Department of Intensive Care Medicine, Amiens Medical Center, Amiens 80054, France

**Keywords:** acute kidney injury, uremic toxins, cardiovascular dysfunction, indoxyl sulfate, para-cresyl sulfate, indole-3-acetic acid

## Abstract

Acute kidney injury (AKI) is a frequent disease encountered in the hospital, with a higher incidence in intensive care units. Despite progress in renal replacement therapy, AKI is still associated with early and late complications, especially cardiovascular events and mortality. The role of gut-derived protein-bound uremic toxins (PBUTs) in vascular and cardiac dysfunction has been extensively studied during chronic kidney disease (CKD), in particular, that of indoxyl sulfate (IS), para-cresyl sulfate (PCS), and indole-3-acetic acid (IAA), resulting in both experimental and clinical evidence. PBUTs, which accumulate when the excretory function of the kidneys is impaired, have a deleterious effect on and cause damage to cardiovascular tissues. However, the link between PBUTs and the cardiovascular complications of AKI and the pathophysiological mechanisms potentially involved are unclear. This review aims to summarize available data concerning the participation of PBUTs in the early and late cardiovascular complications of AKI.

## 1. Introduction

Acute kidney injury (AKI) is defined as the sudden loss of the kidney’s excretory function, characterized by an increase in serum creatinine concentrations and/or a decrease in urine output. The Kidney Disease Improving Global Outcomes (KDIGO) criteria classify AKI severity in three stages based on changes in serum creatinine levels and urine output [1]. The prevalence of AKI is in constant progression, particularly in intensive care units (ICU) [2,3,4]. Recent studies estimated that AKI affects approximately one in five hospitalized adults and one half of those admitted to the ICU [5,6], in particular, the elderly and patients with comorbidities, such as chronic kidney disease (CKD), diabetes mellitus, and cardiovascular disease. AKI is associated with a poor outcome, with estimated hospital mortality of 20% in high-income countries, which increases with AKI severity [5,7]. AKI is frequently associated with remote organ dysfunction, particularly, in critically ill patients, with a mortality rate that increases with the number of failing organs [8]. The impact of one episode of AKI, even short-lived, has been associated in several studies with a high risk of developing CKD and mortality [9,10,11]. Indeed, AKI generates an acute uremic state that results in not only electrolyte derangement and the disruption of volume homeostasis but also the accumulation of metabolic waste, which can induce cell and tissue damage [12]. Gut-derived protein-bound uremic toxins (PBUTs), especially indoxyl sulfate (IS), para-cresyl sulfate (PCS), and indole-3-acetic acid (IAA), are gut–microbiota metabolites that accumulate in the blood when the excretory function of the kidneys decreases and are not efficiently cleared following renal replacement therapy. Simultaneously, the accumulation of gut-derived PBUTs may be enhanced by kidney disease-associated dysbiosis [13]. PBUTs and their role in the cardiovascular complications of CKD have been extensively studied [14]. However, PBUTs may also generate early tissue damage and thus have deleterious effects on the cardiovascular system during AKI. The purpose of this review article is to summarize the available literature related to the cardiovascular complications of AKI and the potential role of PBUTs.

## 2. Cardiovascular Complications in AKI Patients

### 2.1. Cardiovascular Complications during an AKI Episode

Early systemic complications of AKI generally include volume overload and electrolyte and acid-base disturbances, particularly hyponatremia, hyperkalemia, and metabolic acidosis. These AKI complications may directly or indirectly alter the vascular and cardiac function and induce tissue damage, such as myocardial injury, by neuroendocrine, inflammatory, or hemodynamic mechanisms [15,16]. It is often difficult, however, to differentiate complications related to AKI per se from those related to the underlying cause of AKI. Indeed, a cardiorenal syndrome (CRS) with a complex pathological interplay between the kidneys and the cardiovascular system can occur during AKI. CRS type 3 comprises situations in which AKI precipitates and/or contributes to the development of acute cardiac injury and/or dysfunction, such as acute decompensated heart failure (ADHF), acute myocardial infarction (MI), and cardiac arrhythmias [17]. Although CRS type 3 is well characterized, there is a paucity of data on its incidence and prevalence, provided that most published studies have primarily focused on the late cardiovascular complications of AKI after hospital discharge or have considered AKI as a complication in selected cardiovascular settings, such as MI [18,19], ADHF [20], coronary angiography [21], or stroke [22]. Nevertheless, AKI is becoming increasingly common worldwide, affecting one in four patients hospitalized for cardiac disease, and appears to be a major contributor to morbi-mortality [16]. Mechanisms linking AKI and cardiovascular complications have been proposed [23], although the course of the pathophysiological events is still poorly understood. The uremic state includes the release of pro-inflammatory cytokines that impair the viability of endothelial and cardiac cells and induce leukocyte infiltration [24,25]. Activation of the renin-angiotensin-aldosterone system induces acute pressure overload and promotes vascular and cardiac remodeling and the development of fibrosis [26,27]. A role for galectin-3, a β-galactoside-binding lectin that plays an important role in cell survival, has also been described in cardiac diastolic dysfunction, inflammation, and fibrosis after AKI [28]. However, the complex interaction between the kidneys and cardiovascular system, in which they interact with each other and share common pathophysiological mechanisms, makes it difficult to establish how an acute uremic state can contribute to cardiovascular dysfunction. Further studies focusing on subsequent early cardiovascular complications during AKI are required to verify this hypothesis.

### 2.2. Cardiovascular Complications after an AKI Episode

The risk of cardiovascular complications and mortality is well established in CKD patients and attributed to the usual (high blood pressure, diabetes, etc.) and less common risk factors (inflammation, PBUTs, etc.) [29,30]. Several clinical studies have also shown a greater risk of late cardiovascular complications after an AKI episode. In a prospective cohort of patients developing AKI within 30 days of cardiac surgery, postoperative AKI was found to be associated with an increased five-year risk of myocardial infarction and heart failure, as well as increased all-cause mortality [31]. Moreover, in a prospective study of 968 patients undergoing cardiac surgery, AKI stage and duration were linked to cardiovascular events during the five years of follow-up [32]. AKI was also shown to be associated with a risk of readmission for heart failure within the first two years of hospital discharge for patients who had experienced an AKI episode, independently of cardiovascular risk factors and a history of heart failure [33,34,35]. Patients who recovered from dialysis-requiring AKI also had a higher long-term risk of global coronary events, major adverse cardiovascular events (MACE) (nonfatal myocardial infarction (MI), cardiac revascularization, and acute ischemic stroke events), and cardiovascular mortality, regardless of subsequent progression to CKD [36,37]. In a meta-analysis that included 254,408 patients, AKI was shown to be associated with a risk of severe cardiovascular events, such as acute myocardial infarction and stroke, and the association was independent of CKD development [38]. Silver et al. also explored the causes of death within the first year of hospital discharge after an AKI episode [39]. Among the 43,422 (28%) patients who died, 28% died from cardiovascular events. Additionally, in 2016, Hsu et al. explored the link between AKI and blood pressure in a cohort of 43,611 patients hospitalized in conventional or intensive care units [40]. They showed that the AKI group was more likely to develop high blood pressure than the non-AKI group. AKI thus appears to have contributed to long-term cardiovascular risk, independently from progression to CKD in these studies. On the contrary, Ikizler et al. studied the association of AKI with cardiovascular events, taking into account the kidney function of patients before and after the episode of AKI [41]. They found that AKI was associated with heart failure and death irrespective of preexisting CKD. However, this association was no longer significant after adjustment for the recovery of kidney function and proteinuria three months after discharge. Similarly, in a cohort of 11,538 patients who developed hospital-acquired AKI, the risk of long-term MACE among patients who fully recovered from AKI was lower than that of patients who did not fully recover [42]. Compared to other studies, the risk of cardiovascular events appeared to be dependent on kidney recovery after AKI in these cohorts.

Concerning these study results, the causal effect of AKI on the occurrence of late cardiovascular complications is still a subject of debate. Notably, patients presenting with AKI are generally older and present predisposing chronic conditions, such as diabetes mellitus, which may, even after adjustment for confounding factors, contribute to the observed greater risk of cardiovascular complications. In addition, a large proportion of the studies focused on selected patients presenting with AKI in a cardiovascular disease setting. These results also highlight the potential influence of the duration and severity of the AKI episode and suggest an impact of the progression from AKI to CKD on the occurrence of late cardiovascular complications after AKI. In this context, the accumulation of PBUTs, particularly in severe and prolonged AKI, may contribute to the observed increased risk of cardiovascular complications through induced endothelial, cardiac, and tubular injury.

## 3. Gut-Derived, Protein-Bound Uremic Toxins and Cardiovascular Dysfunction in Experimental AKI Models

The formation and metabolism of IS, PCS, and IAA, from their precursors (indole for IS and IAA and p-cresol for PCS), produced by gut microbiota, to their kidney excretion through organic anion transporters (OATs) and the mechanisms that lead to their accumulation in kidney disease, have been well described in recent reviews [14,43]. A number of experimental studies have explored the relationship between gut microbiota and the kidney in acute and chronic models, highlighting inter-organ crosstalk [44,45,46]. Kidney failure is indeed responsible for the disturbance of the gut microbiota, and dysbiosis is linked to the progression of kidney failure [13]. However, factors that may influence PBUT accumulation in the gut–kidney axis appear to be different between CKD and AKI. In CKD, external factors associated with a specific diet (low fiber intake), longterm antibiotic treatment, phosphate binder treatment, and iron supplementation, and the internal factor of high urea levels modify the gut microbiota and intestinal barrier permeability [47,48,49,50]. Although AKI and CKD may share common factors, such as a specific diet or the use of antibiotics [51], a reduction in short-chain fatty acid levels in AKI may play a specific role in the formation of PBUTs by favoring an inflammatory state associated with intestinal barrier disruption [13,46,52,53,54,55]. The following sections will focus on the cardiovascular consequences of the main gut-derived PBUTs studied in experimental models of AKI.

### 3.1. Indoxyl Sulfate

Indoxyl sulfate (IS) is a PBUT that strongly contributes to endothelial damage during CKD. IS is known to have many specific deleterious effects on the vascular wall, in particular, by decreasing endothelial relaxation, cell viability, and proliferation and by inducing oxidative stress [56,57,58,59]. In addition, it promotes the expression of adhesion molecules, such as ICAM-1 and MCP-1, which are associated with leukocyte extravasation, and alters endothelial permeability [60,61,62]. Moreover, it is responsible for a pro-thrombotic state by promoting tissue factor production [63,64]. A number of studies have also shown the toxicity of IS for the heart, with cardiac pro-fibrotic, pro-hypertrophic, and pro-inflammatory effects associated with the induction of oxidative stress [65,66,67,68,69,70]. However, in the specific context of AKI, its role in vascular and cardiac dysfunction is poorly understood. A number of studies have examined the vascular effects of IS in vitro using very short-term exposure, which may mimic acute exposure to IS during AKI [58,60,64]. Dou et al. exposed endothelial cells for five hours to four different concentrations of IS and showed an increase in ROS production within one hour of exposure [57]. Moreover, endothelial cells showed reduced viability and NO production after three hours of exposure to IS [59]. An increase in tissue factor and ICAM-1 expression by endothelial cells was also found after two and six hours of IS exposure, respectively [60,63]. Exposure of endothelial progenitor cells (EPCs) to IS was shown to result in a decrease in EPC viability (less proliferation and more senescence and autophagy) and the induction of oxidative stress in a dose-dependent manner. In an animal model of AKI, consisting of a unilateral ischemia-reperfusion model generated by the ligature of the left renal artery for 40 min, IS attenuated eNOs expression in the endothelium of the arteries and ischemic kidney and reduced EPC mobilization from the bone marrow [71]. More recently, two experimental studies highlighted the action of acute IS exposure on the decrease in vasorelaxation in rat aorta linked to a reduction in the release of NO [72,73]. Moreover, Savira et al. explored the role of IS in vascular and cardiac dysfunction [74,75]. They showed that IS induced cardiomyocyte hypertrophy and decreased vasorelaxation by activation of the ASK1 pathway. Furthermore, Shen et al. demonstrated the action of IS on the endothelial expression of E-selectin mediated by IL-1β in an AKI mouse model [76]. E-selectin expression was higher in kidney endothelial cells from AKI mice than in controls. In vitro, E-selectin expression was directly associated with the IS concentration in endothelial cells pre-exposed to IL-1β. These effects were associated with ROS production and higher monocyte adhesion to endothelial cells. As for E-selectin, ICAM-1 expression was induced by IS in endothelial cells pre-exposed to IL-1β in a second study [77]. The acute effect of IS on leukocyte adhesion and extravasation was also confirmed in a rat model exposed to various times of IS infusion [62]. The acute cardiac toxicity of IS was also explored in vitro [78]. The authors demonstrated a dose-dependent increase in cardiomyocyte apoptosis after 24 h of IS exposure. These results were confirmed by animal studies. Shen et al. highlighted cardiac dysfunction with pathological changes in echocardiography parameters associated with higher brain natriuretic peptide (BNP) levels and greater cardiomyocyte apoptosis in AKI mice. In addition, treatment by AST-120, an oral charcoal adsorbent that decreased IS levels, improved all parameters [77]. Furthermore, they evaluated the cardiac effect of EPC treatment in AKI mice [79]. AKI mice infused with EPC showed improved cardiac echocardiography parameters, with less cardiomyocyte apoptosis initially induced by IS and IL-1β. These results were associated with the inhibition of a pro-apoptotic protein by EPC, probably through the decrease in IS and IL-1β concentrations. These studies confirmed the direct role of IS on vascular and cardiac cell toxicity and induction of the pro-inflammatory and oxidative states during AKI. However, the association between IS, cardiovascular dysfunction, and cardiovascular complications can also be explained by the pathological action of IS on kidney disease progression. Indeed, several experimental studies have highlighted the effect of IS on the development of renal fibrosis through oxidative stress, the activation of endoplasmic reticulum stress, and the epithelial-mesenchymal transition [80,81,82,83,84,85].

### 3.2. Para-Cresyl Sulfate

As does IS, para-cresyl sulfate (PCS) shows toxicity towards vascular and cardiac tissues, mainly explored in CKD. It is responsible for endothelial damage, with alterations in endothelial wall permeability, microparticle release, and leukocyte recruitment [62,86,87]. It also acts on the migration and proliferation of vascular smooth muscle cells [88]. Additionally, PCS is directly involved in cardiac diastolic dysfunction by increasing cardiomyocyte apoptosis and ROS production [89]. Similar to IS, it is also responsible for cardiomyocyte hypertrophy and fibroblast collagen synthesis subsequent to the activation of ASK, a regulator of the cellular stress response [74]. P-cresol, the precursor of PCS, also has effects on the endothelium, with a decrease in cell proliferation and the disruption of adherent junctions [58,90]. Only a few experimental studies investigated the role of PCS on vascular and cardiac dysfunction during AKI. Two in vitro studies assessed the stimulatory effect of acute exposure to PCS on leukocytes, showing oxidative burst activity [91,92]. This action was associated with increased leukocyte adhesion to the vascular wall after a short-term infusion of PCS in vivo [62]. Moreover, Gross et al. demonstrated its deleterious effect on vascular reactivity in an ex vivo model of aortic rings exposed to PCS [93]. After short-term exposure to PCS (30 min), the thoracic aorta showed pathological constriction mediated by rho-kinase activation. This effect was associated with ROS production by endothelial and vascular smooth muscle cells in vitro. Moreover, PCS induced higher vascular permeability in rat vessels after 10 to 60 min of exposure at various concentrations, suggesting that PCS can induce endothelial barrier dysfunction [94]. Moreover, Huang et al. explored the effect of various concentrations of PCS on cardiomyoblasts in vitro [95]. After short-term exposure to low-level PCS, the cardiomyoblasts showed less proliferation and mitochondrial hyperfusion. This effect was considered to be a stress-induced response to acute PCS exposure. P-cresol (the gut precursor of PCS before metabolic sulfatation by the liver) was also shown to be responsible for altered cardiomyocyte contractility and the disruption of gap junctions after acute exposure [96]. However, the acute action of PCS in cardiac dysfunction needs to be evaluated in animal studies. As for IS, PCS is also responsible for the progression of renal fibrosis subsequent to the induction of oxidative stress leading to ROS production [85,97,98,99]. Thus, its action on the AKI to CKD transition should also lead to CKD-associated cardiovascular complications.

### 3.3. Indole-3-Acetic Acid

Indole-3-acetic acid (IAA), another PBUT, is also known to have adverse effects on vascular function during CKD. Its main role is to activate aryl hydrocarbon receptor (AhR) signaling pathways, similar to IS [63,100]. Thus, it is responsible for ROS production and pro-inflammatory molecule Cox-2 activation, as well as tissue factor production, in endothelial cells [63,101]. Three experimental studies explored the effect of very short-term exposure of endothelial cells to IAA in vitro and highlighted its pro-inflammatory and pro-apoptotic effect on endothelial cells and their progenitors [63,101,102]. However, these results need to be completed by in vivo studies. Moreover, the potential deleterious actions of IAA on the heart are still unknown. Furthermore, its contribution to the possible transition from AKI to CKD and, thus, to CKD-associated CV complications has not been specifically studied.

## 4. Gut-Derived Protein-Bound Uremic Toxins and Cardiovascular Dysfunctions in Clinical AKI Studies

### 4.1. Gut-Derived Protein-Bound Uremic Toxin Accumulation in AKI Patients

Data on PBUT accumulation in AKI patients are limited, and most of the studies focused on the IS retention profile. The main characteristics of these studies are presented in Table 1. The relative heterogeneity concerning sample size, population background, clinical setting, the timing of measurements, and severity of AKI may limit the interpretation of the data. The kinetics of IS accumulation appear to follow those of serum creatinine [103], and two studies showed that IS levels increase with the severity of AKI [104,105], the highest observed among patients presenting with severe AKI and those who needed renal replacement therapy support. Except for one study reporting a particularly high level [71], the observed level of total IS remained relatively low compared to that in CKD [106], ranging from 0.64 to 3.33 µg/mL. Such relative variability of observed levels could be explained by several factors, notably a change in the composition of the gut microbiota relative to the patients’ genetic background and clinical setting. Indeed, several factors, such as systemic inflammation, parenteral nutrition, antibiotics, or intestinal permeability, may alter the composition of the gut microbiota [51]. On the other hand, systemic inflammation and fluid accumulation, resulting in a decrease in serum albumin levels, may also reduce the measured total serum level of IS, particularly in septic AKI. Two studies analysed the correlation between IS levels and recovery from AKI [104,105], and one study reported an independent association between total IS levels at AKI diagnosis and 90-day mortality [107]. In addition, the complications of AKI patients may depend on the elimination time course of PBUTs, reflecting the AKI recovery rate independently from renal replacement therapy. Indeed, in the study of Veldeman et al., a decrease in IS and PCS concentrations was observed only in the group of patients with a favorable AKI outcome [104]. As indicated previously, gut-derived PBUTs may accumulate, resulting not only from a decrease in kidney excretion but also from kidney-disease-associated dysbiosis. The gut–kidney axis has been investigated in a number of clinical studies, mostly in CKD [108,109,110,111]. In septic AKI, impaired gut barrier permeability, with the translocation of bacterial and inflammatory molecules, has also been shown to be associated with the progression of kidney injury and the AKI to CKD transition [112,113]. However, more clinical data are needed to precisely explore the gut–kidney axis and both PUBT generation and accumulation in AKI.

### 4.2. Association between Gut-Derived Protein-Bound Uremic Toxins and Cardiovascular Outcomes of AKI Patients

**Figure 1:**
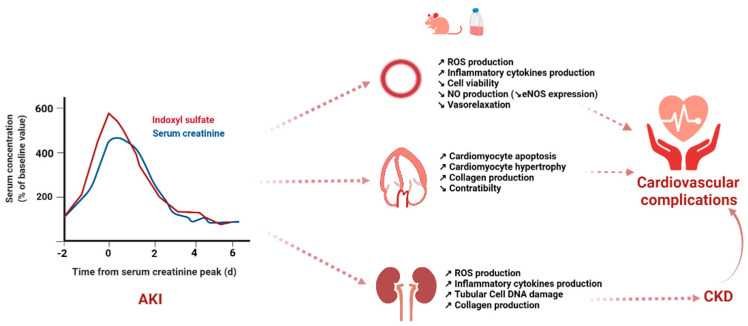
Hypothetical pathophysiological link between acute indoxyl sulfate accumulation and cardiovascular complications after AKI. The serum creatinine and indoxyl sulfate serum concentration-time curves are drawn from data obtained from a patient who developed AKI after cardiac surgery [104]. AKI, acute kidney injury; CKD, Chronic kidney disease; DNA, deoxyribonucleic acid; eNOS, endothelial nitrite oxyde synthase; NO, nitrite oxyde; ROS, reactive oxygen species. Created with BioRender.com。

The role of gut-derived PBUTs in AKI complications is as yet underexplored. A growing body of evidence supports the contribution of altered renal vascular function in the initiation and extension of tubular injury. For example, in a large cohort of cardiac surgery patients, Mansour et al. showed that postoperative elevation of the levels of VEFG and PGF, two proangiogenic markers, was associated with a reduced risk of AKI and death, whereas an elevation in the level of VEGFR1, an antiangiogenic factor, was associated with an increased risk of AKI [114]. Furthermore, EPC infusion improved the ischemic-AKI prognosis in animal models. On the contrary, several clinical studies showed a decrease in the number and impaired function of EPCs in CKD patients. However, data regarding the relationship between acute UT accumulation and cardio-vascular dysfunction are limited to a study involving 41 patients who developed post-cardiac surgery AKI [71]. In this study, Wu et al. showed a negative correlation between IS levels and the number of circulating EPCs. However, this negative correlation was only observed for an IS concentration > 51.16 µg/mL, which is up to 10-fold higher than that observed in other clinical studies in AKI settings, and no data on the clinical outcome were available in this experimental study. Indeed, most clinical studies have explored the relationship between IS levels and mortality and/or short-term progression of AKI. As suggested in animal models, the deleterious tubular effects of acute PBUT accumulation during AKI may also precipitate the development of or progression to CKD and thus indirectly favor the occurrence of cardiovascular complications [85]. Clinical studies on the long-term renal and cardiovascular effects of UT accumulation during AKI are lacking, and this topic merits further research. Figure 1 summarizes the potential physiopathological link between acute IS accumulation and cardiovascular complications after AKI.

## 5. Conclusions

There is much evidence for the cardiovascular toxicity of PBUTs, and their association with cardiovascular events and mortality during CKD has been extensively studied. Data showing that PBUTs can also accumulate in the blood during AKI are now available, although the levels reported appear to be lower than those observed in CKD. Additionally, experimental studies strongly suggest that short-term exposure to PBUTs can be sufficient to produce deleterious vascular and cardiac effects. Among gut-derived PBUTs, IS has been the most extensively evaluated in AKI, showing structural damage (pro-apoptotic effect and oxidative stress on endothelial and cardiac cells) and a functional impact (impairment of vasoreactivity and alteration of diastolic cardiac parameters) in vitro and in vivo. Furthermore, IS toxicity is also related to the release of pro-inflammatory cytokines and the expression of adhesion molecules, which may contribute to increased susceptibility to sepsis complications in critically ill patients. Although the pathological effects of PCS have been less well described, it acts equally on vasoreactivity impairment and endothelial and cardiac cell viability. In clinical studies, the level of PBUTs, mostly that of IS, appear to correlate with short-term mortality [107], but with several confounding factors, in particular, in cases of sepsis. Nevertheless, available data concerning the participation of PBUTs in the early and late cardiovascular complications of AKI are relatively scarce compared to those on CKD, resulting in the lack of a strong argument, and justifies specific additional experimental and clinical studies. The use of PBUT kinetics (concentration, amplitude, and duration) during AKI as a prognostic marker in the management of AKI patients also requires further exploration. Until the link between PBUT levels and the CV complications of AKI have been formally established, post-AKI therapeutic management will continue to be based on the standard recommendations for controlling CV risk, consisting of monitoring, which could be reinforced (or at least considered) independently of the establishment of CKD [115].

## Figures and Tables

**Table 1:** Observational studies of the effects of PBUT accumulation in AKI patients.

Authors (Year) Country	Setting	No. of Patients	Staging of AKI	Measurement	Uremic Toxin Level (µg/mL)	Main Results
Wu et al. (2013) [71] Taiwan	Post-cardiac surgery AKI	41	AKIN Stage 1: 17 (41.4) Stage 2: 12 (29.3) Stage 3: 12 (29.3)	*t*IS at AKI diagnosis	*t*IS mean ± SD 28.78 ± 20.04	Negative correlation between tIS level above 51.16 µg/mL and the human endothelial progenitor cell count.
Veldeman et al. (2019) [104] Belgium	Sepsis in ICU Septic shock (63%)	194	RIFLE No AKI: 64 (33.0) Risk: 40 (20.6) Injury: 57 (29.4) Failure: 33 (17.0)	*t*IS and *t*PCS at admission (D0) and Dend (D4 or day before drop-out)	*t*IS at D0 median [IQR] No AKI: 0.258 [0.097–0.610] AKI: 0.64 [0.252–1.802] -Risk: 0.377 [0.231–0.908] -Injury: 0.50 [0.169–1.716] -Failure: 1.785 [0.762–3.400]	-Correlation between severity of AKI and *t*IS level. -Decrease in *t*IS in all AKI groups between D0 and Dend. -No change in tIS level observed between D0 and Dend in cases of worsening of kidney function, but it decreased in cases of recovery.
Wang et al. (2019) [107] China	Hospital-acquired AKI	262	KDIGO Stage 1: 119 (45.5) Stage 2: 63 (24.0) Stage 3: 80 (30.5)	*t*IS at baseline, AKI diagnosis (n = 262) and 7 days after (n = 89)	*t*IS at AKI diagnosis mean ± SD 2.7 ± 0.8	*t*IS > 2.74 µg/mL was associated with an increased Day 90 mortality rate with an aHR [95%CI] of 2.92 [1.76–4.86] *p* < 0.001.
Andre et al. (2020) [103] France	Post-cardiac surgery AKI	8		Time course of *t*IS, *t*PCS, and *t*IAA serum concentration according to that of creatinine	Peak concentration (min–max) tIS: 1.52 (0.35–2.62) tPCS: 9.33 (4.30–16.00) tIAA: 0.97 (0.60–1.80)	Serum creatinine-like accumulation and elimination profiles
Selim et al. (2021) Egypt [105]	Toxic AKI in ICU	74	RIFLE Risk: 15 (20.3) Injury: 28 (37.8) Failure: 31 (41.9)	*t*IS and *f*IS within 48 h after toxic AKI, then at weeks 1 and 2 (or as ended earlier)	Basal *t*IS median [range] * tIS 3.33 [0.00–37.31]	Correlation between AKI severity and IS level. Association between basal IS level and AKI recovery at discharge No association between IS level and in-hospital mortality

* estimated according to Figure 3 in reference [105]. aHR: adjusted hazard ratio, IAA: indol-3-acetic acid, IS: indoxyl sulfate, PCS: para-cresyl sulfate, tIS: total IS, fIS: free IS.

## Data Availability

Not applicable.
